# Physician Burnout and the Electronic Health Record Leading Up to and During the First Year of COVID-19: Systematic Review

**DOI:** 10.2196/36200

**Published:** 2022-03-31

**Authors:** Clemens Scott Kruse, Michael Mileski, Gevin Dray, Zakia Johnson, Cameron Shaw, Harsha Shirodkar

**Affiliations:** 1 School of Health Administration College of Health Professions Texas State University San Marcos, TX United States

**Keywords:** electronic health record, physician burnout, quality improvement, psychiatry, medical informatics, COVID-19, pandemic, health informatic, health care, health care professional, health care infrastructure, health care system, mental health, cognitive fatigue

## Abstract

**Background:**

Physician burnout was first documented in 1974, and the electronic health record (EHR) has been known to contribute to the symptoms of physician burnout. Authors pondered the extent of this effect, recognizing the increased use of telemedicine during the first year of COVID-19.

**Objective:**

The aim of this review was to objectively analyze the literature over the last 5 years for empirical evidence of burnout incident to the EHR and to identify barriers to, facilitators to, and associated patient satisfaction with using the EHR to improve symptoms of burnout.

**Methods:**

No human participants were involved in this review; however, 100% of participants in studies analyzed were adult physicians. We queried 4 research databases and 1 targeted journal for studies commensurate with the objective statement from January 1, 2016 through January 31, 2021 (n=25).

**Results:**

The hours spent in documentation and workflow are responsible for the sense of loss of autonomy, lack of work-life balance, lack of control of one’s schedule, cognitive fatigue, a general loss of autonomy, and poor relationships with colleagues. Researchers have identified training, local customization of templates and workflow, and the use of scribes as strategies to alleviate the administrative burden of the EHR and decrease symptoms of burnout.

**Conclusions:**

The solutions provided in the literature only addressed 2 of the 3 factors (workflow and documentation time) but not the third factor (usability). Practitioners and administrators should focus on the former 2 factors because they are within their sphere of control. EHR vendors should focus on empirical evidence to identify and improve the usability features with the greatest impact. Researchers should design experiments to explore solutions that address all 3 factors of the EHR that contribute to burnout.

**Trial Registration:**

PROSPERO International Prospective Register of Systematic Reviews CRD42020201820; https://www.crd.york.ac.uk/prospero/display_record.php?RecordID=201820

**International Registered Report Identifier (IRRID):**

RR2-10.2196/15490

## Introduction

### Background

This systematic review examined the state of physician burnout incident to the electronic health record (EHR), compounded by the stress of managing the pandemic in the first year of COVID-19. Neither physician burnout nor the EHR are new; however, the additional stress of managing a pandemic may make the relationship between these 2 variables clearer. The clinical psychologist Herbert Freudenberger [1] is attributed to the first mention of physician burnout in 1974, as he observed physician interaction in the drug-addled East Village of New York City. His description of burnt-out physicians mirrored the physicians’ description of burnt-out patients with drug addiction in terms of a feeling of disassociation as depicted by the definition in the following sections. Physician burnout can be detrimental to physician well-being and to the quality of care provided and can result in higher turnover [2-4]. It is a significant problem that has been attributed to the EHR.

### Rationale

The EHR has become a pervasive entity in the lives of all health care workers. Very few processes in the health care field are independent of the EHR. This “digital version of the patient’s chart is a real-time, patient-centered record that makes information available instantly and securely to authorized users” [5]. Physician burnout is “a long-term stress reaction marked by emotional exhaustion, depersonalization, and a lack of sense of personal accomplishment” [6]. Physician burnout was already identified as a worldwide health issue before COVID-19, and digital tools such as the EHR are cited as a contributing factor to this issue [7,8]. Factors associated with the EHR cited in relation to physician burnout are usability, workflow, and documentation time [8-13]. The documentation inherent to the EHR requires significant time, as much as 2:1 hours of direct clinical face-to-face time and as much as 2 hours outside of office hours [14]. Some authors list burn-out as a new pandemic and a new normal [15,16].

A systematic review of 182 studies on a similar topic was conducted in 2018. It examined physician burnout data over a 17-year period. It identified a high incidence of physician burnout, but it failed to attribute the EHR as a contributor [17]. Another systematic review of 50 studies was conducted in 2019. It identified 4 interventions (teamwork, time management, transitions, and technology) to assuage the effects of physician burnout [10]. A systematic review in 2020 of 81 studies found interventions to decrease the digital-tool burden (training, reduced documentation and task time, expanded care teams, leveraged quality improvement and processes in workflows) in 68% of articles analyzed [9].

### Objectives

The purpose of this research was to examine physician burnout issues incident to the EHR prior to and during the first year of the COVID-19 pandemic by analyzing the literature from the last 5 years. We defined physician burnout as emotional exhaustion, depersonalization, and lack of sense of personal accomplishment [6]. We examined facilitators and barriers to the adoption of mitigation strategies of burnout incident to the EHR.

## Methods

### Protocol and Registration

Authors of this systematic review followed the protocol by Kruse [18] for conducting a systematic review and reported results in accordance with PRISMA (Preferred Reporting Items for Systematic Reviews and Meta-Analysis) [19]. The research was registered with PROSPERO on August 31, 2020.

### Eligibility Criteria

To be eligible for this study, articles had to be published in English in peer-reviewed, academic journals between January 2016 and January 2021. All study designs were accepted including both quantitative and qualitative studies with humans of all ages; however, other systematic reviews were excluded from the selection.

### Information Sources

On January 29, 2021, we used a standard search string to query 4 databases: PubMed (MEDLINE), CINAHL (exclude MEDLINE), Web of Science, and Science Direct. We also performed a journal-specific search of the Mayo Clinic Proceedings.

### Search Strategy

We created a Boolean search string to combine key terms listed in the Medical Subject Headings (MeSH) of the US Library of Medicine [(“electronic health record” OR “electronic medical record”) AND (“physician burnout”) AND COVID-19]. We used the same search strategy in all databases. We used similar filter strategies in each database, because not all databases offer the same tools.

### Study Selection Process

In accordance with the protocol by Kruse [18], we searched key terms in all databases, filtered results, and screened abstracts for applicability. Reviewers rejected articles if they did not produce results (were not research), such as protocols, opinions, or did not address physician burnout and use of the EHR.

### Data Collection Process

We used an Excel spreadsheet as a data extraction tool, collecting additional data at each step of the process. This spreadsheet was standardized in the protocol by Kruse [18]. We used a series of 3 consensus meetings. The first consensus meeting was held after abstract screening. Subsequent consensus meetings identified observations and themes.

### Data Items

In accordance with the protocol by Kruse [18], we collected the following fields of data at each step: PICOS (participants, intervention, results compared to the control group, health outcomes, study design), bias, effect size, country of origin, statistics used, strength of evidence, quality of evidence, and 3 data fields specific to the objective of this systematic review (patient satisfaction, barriers, and facilitators). Data items and observations became the subject of the second and third consensus meetings.

### Risk of Bias Assessment and Reporting

We observed bias and assessed the quality of each study using the Johns Hopkins Nursing tool for Evidence Based Practice (JHNEBP) [20]. We considered the instances of bias in how to interpret the results because bias can limit external validity.

### Effect Measures

Because we accepted mixed methods and qualitative studies, we were unable to standardize summary measures as would be performed in a meta-analysis. Effect size was not reported in any study of the group for analysis.

### Synthesis Methods

During the screening process, reviewers compared elements of the abstract against the objective statement of this review. Article abstracts that matched our objective statement were marked for inclusion. The rest of this subheading is for meta-analyses—not for systematic reviews. Although the protocol by Kruse [18] for conducting a systematic review uses elements of a meta-analysis, it falls short of this standard.

### Additional Analyses

We performed a narrative analysis of the observations to convert them into themes (common threads between articles) [21]. We calculated a frequency of occurrence and reported these in a series of affinity matrices. This technique does not imply a level of importance of these observations, but instead, it simply illustrates the probability of occurrence of these observations across the group for analysis.

## Results

### Study Selection

**Figure 1:**
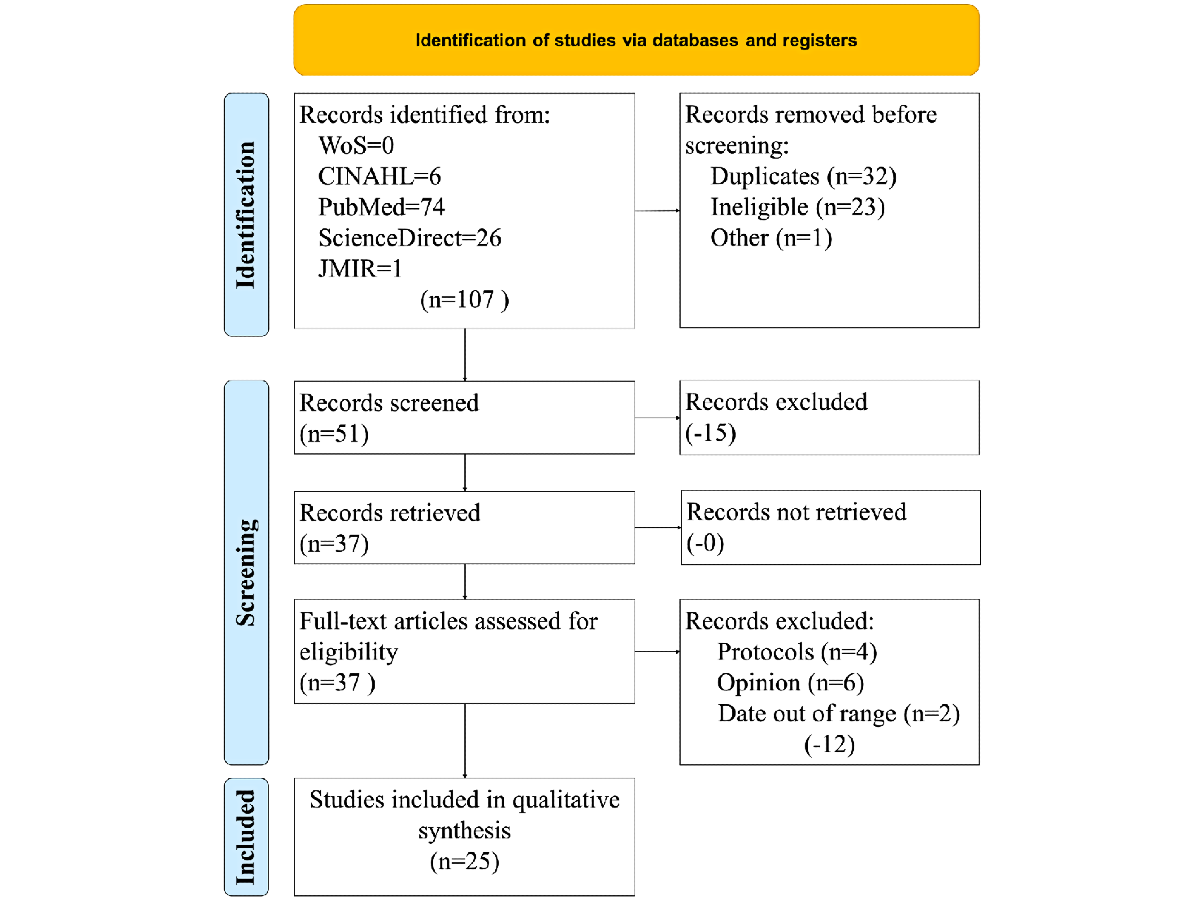
Study selection process. JMIR: Journal of Medical Internet Research; WoS: Web of Science.

Figure 1 illustrates our study selection process from the 4 databases and 1 targeted journal search. A kappa statistic was calculated based on levels of agreement between reviewers (k=0.64, moderate agreement) [22,23].

### Study Characteristics

In accordance with PRISMA 2020, a PICOS table was created from the group of articles analyzed (see Table 1). Of the 25 articles analyzed over the 5-year period, 100% of the participants were adult physicians, and all studies used the EHR as at least one of their foci in their study. Interventions ranged from using the EHR to implementing EHR training or physician partners or scribes. Results varied across studies. Many researchers found training, education, scribes, or physician partners significantly reduced symptoms of physician burnout. Additional explanation of these results will be provided below. Interventions to reduce physician burnout noted improvements in physical pain and psychological outlook. More than half (13/25, 52%) of the study designs were qualitative in nature. Studies are ordered as most recent to oldest: 2021 (n=2) [24,25], 2020 (n=4) [11,26-28], 2019 (n=6) [29-34], 2018 (n=8) [35-42], 2017 (n=2) [43,44], and 2016 (n=2) [45,46].

The 25 studies examined physician burnout with some intervention of the EHR before and during the COVID-19 pandemic. Of the 25 studies, 13 (52%) were qualitative studies, 4 (16%) were mixed methods, 2 sets of 2 (16%) were pre-post or observational, and 3 individual studies (12%) were cross-sectional, cohort, or a meta-analysis. Either scribes or physician partners to enter data into the EHR during the encounter were used in 2 studies [40,46]. This intervention resulted in a decrease in symptoms of physician burnout with zero effect on patient satisfaction. EHR training or a sprint improvement process (customizing local tools) was used by 2 studies to help physicians become more efficient with the EHR [33,41]. These studies also saw a decrease in symptoms of physician burnout with zero effect on patient satisfaction.

**Table 1:** PICOS (participants, intervention, results [compared with a control], outcome, and study design) characteristics of the included studies.

Authors	Participants	Intervention	Result themes	Medical outcome themes	Study design
Hu et al [24]	Adult health care professionals in the ICU^a^ (1122 or 46.54% doctors, 1289 or 53.46% nurses)	EHR^b^	Low frequency of exercise, comorbidities, high-quality hospital has high expectations, more night shifts, longer on the job, few paid vacations	None reported	Qualitative
Rialon et al [25]	Adult health care professionals in pediatrics (68% male, 84% White, 42-60 years old)	EHR	Long hours or workload, no time for themselves, poor work-life balance, loss of autonomy, poor relationships with colleagues	None reported	Qualitative
Giess et al [27]	Adult nonradiologists and radiologists	EHR	Radiologists more likely to report symptoms of burnout	None reported	Qualitative
Kinslow et al [28]	Adult health care professionals (41, 50.6% identified as male; 39, 48.1% identified as female; 1, 1.2% preferred not to answer; 62, 76.5% reported being a resident in a community teaching hospital; 19, 23.5% reported being a resident in a university hospital	EHR	Women at higher risk of burnout and more likely to report suicidal ideations, poor work-life balance, long hours or workload, community-affiliated residents more likely to report suicidal ideation	None reported	Qualitative
Anderson et al [26]	Adult family medicine trainees (postgraduate years 1 through 3) and 10 family medicine faculty at the University of Arizona College of Medicine-Phoenix Family Medicine Residency	EHR	Long hours or workload	None reported	Observational
Khairat et al [11]	Adult physicians completing an EHR simulation activity, 52% female, mean age 33.2 years	EHR	Cognitive fatigue, design issues	Physical fatigue, cognitive weariness	Cross-sectional
Murphy et al [31]	Adult physicians (68% primary care physicians, 32% specialists) at 6 large health care organizations using 4 different EHR systems	EHR	Message complexity, design issues, cognitive fatigue, poor relationships with colleagues, message content	None reported	Qualitative
Tran et al [34]	Adult faculty physicians at 10 university-affiliated primary care clinics; survey sent to 190 faculty members and completed by 107 (56%) providers (86 physicians [MD/DO], 19 advanced practice providers [NP/PA], 2 providers who declined to answer the question); women = approximately two-thirds of the survey respondents; majority of the providers trained in family medicine (57%), internal medicine (27%), or pediatrics (18%)	EHR	Long hours or workload, poor work-life balance	None reported	Qualitative
Gardner et al [29]	Adult practicing physicians in Rhode Island	EHR	EHR-related or work-related stress	Work stress	Qualitative
Kroth et al [30]	Adult ambulatory primary care and subspecialty clinicians from 3 institutions (85.5% physicians, 56.7% women, 68.4% worked in primary care)	EHR	Design issues, lack of interoperability, poor work-life balance, seated position caused problems with back or wrist pain and posture	Posture, back pain	Qualitative
Sieja et al [33]	Adult clinicians in endocrinology, neurology, hematology, obstetrics, and gynecology as well as advanced practice providers	EHR Sprint process improvement	Long hours or workload	None reported	Pre-post
Quinn et al [32]	Adult physicians with an EHR	EHR	Design issues	None reported	Mixed methods
Robinson and Kersey [41]	Adult physicians from 30 specialties completing a total of 46 trainings from 2014 to 2016	EHR training	EHR improves quality and safety, readability, clinical workflow, and accuracy of documentation; efficiency gains with training; system speed and reliability issues	None reported	Mixed methods
Pozdnyakova et al [40]	Adult faculty and a convenience sample (n=325) of their patients at an academic clinic (of patients: 69% Black, 65% female, 48% >65 years old); 373 patients completed surveys; 48 (13%) excluded due to incomplete data, and 325 analyzed (166 scribed and 159 nonscribed visits; Figure 1)	Scribes to assist with EHR workload	Long hours or workload	None reported	Pre-post
Marmor et al [39]	Adult physicians of internal medicine, cardiology, and gastroenterology	EHR	Time spent in EHR affects patient satisfaction	None reported	Meta-analysis
Denton et al [35]	Adult physicians at 2 urban emergency departments	EHR	EHR improves clinical workflow, door-to-doctor and time to decision, and quality and safety	None reported	Qualitative
Kroth et al [38]	Adult clinicians from 2 focus groups at 3 health care facilities with different EHRs (71% women, 98% physicians, 73% worked in primary care for an average of 11 years)	EHR	Long hours or workload, EHR-related or work-related stress, poor work-life balance	Eye strain, hand or wrist pain, back pain	Qualitative
Hauer et al [36]	Adult member and nonmember physicians practicing in Wisconsin whose email address is listed in the Wisconsin Medical Society’s database	EHR	Loss of autonomy, poor relationships with colleagues, loss of autonomy, poor work-life balance	None reported	Qualitative
Young et al [42]	Adult family physician attendings, residents, and their ambulatory patients in 982 visits in clinics affiliated with 10 residencies of the Residency Research Network of Texas	EHR	Long hours or workload	None reported	Observational
Khairat et al [37]	Adult ED physicians at a large tertiary academic hospital, 50% female, 43% residents, 57% attendings	EHR	Design issues, long hours or workload, system speed or reliability issues	None reported	Mixed methods
Arndt et al [47]	Adult family medicine physicians in a single system in southern Wisconsin (100% Epic users; 43% female)	EHR	Long hours or workload	None reported	Cohort
Shahmoradi et al [44]	Adult workforce at 15 ambulatory hospitals (67% female, 75.05% with at least a BSc degree, 45.5% with age of 31-41 years, 46.67% employed <15 years)	EHR	Design issues	None reported	Qualitative
Gregory et al [43]	Adult primary care physicians at a large medical center	EHR alerts	Alert fatigue, cognitive fatigue	Physical fatigue, cognitive weariness	Mixed methods
Jamoom et al [45]	Adult physicians	EHR	Long hours or workload, longer on the job	None reported	Qualitative
Reuben et al [46]	Adult physicians were surveyed, including the pilot physicians and others who had experienced ≥1 session with a physician partner	Physician partners to help with EHR workload	Scribes or physician partners can decrease symptoms of burnout.	None reported	True experiment

^a^ICU: intensive care unit.

^b^EHR: electronic health record.

### Risk of Bias Within and Across Studies

The JHNEBP quality assessment tool was used to identify the strength and quality of evidence in the literature. These are illustrated in Table 2. Of the articles, 80% (20/25) had a strength of III, and 88% (22/25) were quality B. This means a vast majority of articles were qualitative, mixed methods, nonexperimental, or quasi-experimental in nature, but their quality was still strong. Regarding the strength of evidence, level I studies were randomized controlled trials or true experiments. Level II studies were quasi-experimental in nature (no randomization). Level III studies were nonexperimental studies or qualitative studies. We did not accept any studies with a strength of evidence lower than III because these categories are opinion rather than research. Regarding the distribution of the 3 levels of evidence quality, in quality category A, research shows consistent results with sufficient sample sizes, adequate controls, and definitive conclusions. In quality category B, research shows reasonably consistent results, sufficient sample sizes, some control, and fairly definitive conclusions. As illustrated, we did not encounter any studies with a quality rating of C.

**Table 2:** Summary of strength and quality of evidence identified with the Johns Hopkins Nursing tool for Evidence Based Practice (JHNEBP; n=25).

Assessment		Frequency, n
**Strength of evidence**		
	I	2
	II	3
	III	20
**Quality of evidence**		
	A	3
	B	22
	C	0

### Results of Individual Studies

Reviewers independently recorded observations for each article commensurate with the objective statement. A thematic analysis was conducted to make sense of the data. When an observation was identified more than once, it became a theme. Themes were created to summarize the observations, but they did not always exactly match the observations. These themes can be observed in Table 3. Articles are sorted by most recent to oldest. Multimedia Appendix 1 and Multimedia Appendix 2 show the observation-to-theme match. Multimedia Appendix 3 shows additional data extracted from each study.

Reviewers conducted a thematic or narrative analysis. Part of this analysis was making sense of the data. When an observation reoccurred, it became a theme. Observations without reoccurrence were just observations. Patient satisfaction, barriers, and facilitators were explored under additional analysis. Scribes and physician partners were used in 3 studies to enter data into the EHR during an appointment, but only 2 of the studies reported on patient satisfaction outcomes.

**Table 3:** Summary of the analysis, sorted most recent to oldest.

Authors	Patient satisfaction themes	Barrier themes	Facilitator themes
Hu et al [24]	EHR^a^ time in clinic negatively affects patient satisfaction; patient dissatisfaction negatively affects doctor-patient relationship; patient dissatisfaction negatively affects physician burnout.	Not reported	Exercise relieves symptoms of burnout; annual vacation relieves symptoms of burnout.
Rialon et al [25]	Not reported	Excessive hours spent in the EHR affect work-life balance, excessive hours spent in the EHR exacerbates symptoms of physician burnout, administrative time in the EHR takes time away from clinic and patients.	Focus on mission of care relieves symptoms of burnout.
Giess et al [27]	Not reported	EHR does not help coordinate care.	Not reported
Kinslow et al [28]	Not reported	Excessive hours spent in the EHR exacerbate symptoms of physician burnout.	Small group sessions
Anderson et al [26]	Not reported	Excessive hours spent in the EHR exacerbate symptoms of physician burnout.	Not reported
Khairat et al [11]	Not reported	EHR must undergo redesign, high number of clicks per process is inefficient.	Not reported
Murphy et al [31]	Not reported	The administrative overhead of the EHR is not conducive to efficient workflow, excessive hours spent in the EHR affect work-life balance, administrative overhead of the EHR is not conducive to efficient workflow.	Local customization (eg, templates, menus) improves efficiency, localized workflow redesign relieves symptoms of burnout.
Tran et al [34]	Not reported	Excessive hours spent in the EHR exacerbate symptoms of physician burnout.	Not reported
Gardner et al [29]	Not reported	Administrative time in the EHR takes time away from clinic and patients, excessive hours spent in the EHR affect work-life balance.	Not reported
Kroth et al [30]	Not reported	EHR must undergo redesign, excessive hours spent in the EHR exacerbate symptoms of physician burnout, high number of clicks per process is inefficient, administrative time in the EHR takes time away from clinic and patients, excessive hours spent in the EHR affect work-life balance.	Not reported
Sieja et al [33]	Not reported	Administrative overhead of the EHR is not conducive to efficient workflow.	Local customization (eg, templates, menus) improves efficiency.
Quinn et al [32]	Not reported	EHR reliability and speed, some patient information is not available due to lack of interoperability, EHR must undergo redesign.	Training increases efficiency.
Robinson and Kersey [41]	Not reported	EHR training takes time away from the clinic.	Institutional endorsement of EHR increases user acceptance of EHR, training increases efficiency.
Pozdnyakova et al [40]	Patient satisfaction not affected by scribe or physician partner in clinic during exam	Some patients do not like scribes or physician partners in the exam room, excessive hours spent in the EHR exacerbate symptoms of physician burnout.	Presence of scribe or physician partner relieves symptoms of burnout, localized workflow redesign relieves symptoms of burnout.
Marmor et al [39]	Time of day affects patient satisfaction more than time spent with patient.	Excessive hours spent in the EHR exacerbate symptoms of physician burnout.	Localized workflow redesign relieves symptoms of burnout.
Denton et al [35]	Not reported	EHR must undergo redesign, high number of clicks per process is inefficient, administrative overhead of the EHR is not conducive to efficient workflow.	EHR increases safety, decreases admission decision time, and decreases length of stay.
Kroth et al [38]	Not reported	EHR must undergo redesign, EHR reliability and speed, some patient information is not available due to lack of interoperability, administrative overhead of the EHR is not conducive to efficient workflow.	Training increases efficiency, presence of scribe or physician partner relieves symptoms of burnout.
Hauer et al [36]	Not reported	EHR must undergo redesign, lack of supporting practice environment, EHR creates a loss of autonomy, excessive hours spent in the EHR affects work-life balance.	Not reported
Young et al [42]	Not reported	Administrative time in the EHR takes time away from clinic and patients.	Not reported
Khairat et al [37]	Not reported	EHR must undergo redesign, EHR reliability and speed.	Not reported
Arndt et al [47]	Not reported	EHR must undergo redesign, excessive hours spent in the EHR affect work-life balance, administrative overhead of the EHR is not conducive to efficient workflow.	Not reported
Shahmoradi et al [44]	Not reported	EHR reliability and speed, excessive hours spent in the EHR exacerbate symptoms of physician burnout, some patient information is not available due to lack of interoperability, administrative overhead of the EHR is not conducive to efficient workflow, EHR investment inhibits short-term profit, EHR must undergo redesign, no standardized vocabulary.	EHR enables rapid access to information, decreases duplicate testing, increases speed of delivery of care, increases accuracy of documentation, increases safety, enables computerized analysis and interpretation of data.
Gregory et al [43]	Not reported	EHR must undergo redesign, administrative overhead of the EHR is not conducive to efficient workflow.	Not reported
Jamoom et al [45]	Not reported	Not reported	Level of physician experience with EHR increases perceived usefulness of EHR
Reuben et al [46]	Patient satisfaction not affected by scribe or physician partner in clinic during exam	Scribes or physician partners cost more money.	Presence of scribe or physician partner relieves symptoms of burnout.

^a^EHR: electronic health record.

### Additional Analysis

Themes and individual observations were organized into tables to reflect the probability of their occurrence in the group for analysis. These affinity matrices are shown and discussed in the following sections. In the interest of saving space, only those with the greatest number of occurrences will be discussed in detail.

### Study Results

Table 4 summarizes the study results observed: 12 themes and 20 individual observations were identified by the reviewers for a total of 68 occurrences in the literature.

Of 68 occurrences, 13 (19%) identified longer hours worked and increased workload as a result of using the EHR. Researchers noted respondents to surveys worked 60-80 hours per week: The extra time was largely attributed to the EHR [25,45]. Physicians spent between 17 minutes and 217 minutes per patient in the EHR, resulting in up to 33 hours per month in the EHR after work hours: These longer hours were highly attributable to symptoms of burnout [26,34]. The nonintuitive nature of the EHR negatively impacted efficiency and contributed to the longer hours [37]. This point leads to the next item most often cited: design issues. This point occurred in 7 of 68 (10%) occurrences. Observations about design were attributed to the user interface, the long length of cut-and-paste notes required, communication and inefficient data-sharing processes, and the requirement to memorize menu and button names [11,30-32,37,44]. The long hours spent in the EHR created a poor work-life balance [25,28,30,34,36,38]. This point occurred in 6 of 68 (9%) occurrences. Many providers felt compelled to complete administrative work in the EHR from home so that they could at least be near their families while completing their workload, but this habit created tension in the household and overall impeded attempts at work-life balance. Four themes occurred 3 times (12%): EHR improves quality and safety [35,41], a general loss of autonomy [25,36], poor relationships with colleagues [25,31,36], and cognitive fatigue [11,31,43]. The increase in quality and safety appeared in the form of greater readability of notes, increased accuracy of clinician notes, a decrease in medical errors, increased clinical efficiency, and ease of data retrieval. Loss of autonomy occurred in the literature as a general lack of control over one’s schedule. Poor relationships with colleagues occurred as lack of team communication, lack of supportive practice environment, and lack of time available in the clinic to build relationships. Cognitive fatigue was only subjectively queried in 1 of the 3 studies: The other 2 were objectively measured as pupillometry and a cognitive weariness index. These themes comprised 60% of the observations. Some of these themes will appear again as either facilitators or barriers to the use of the EHR to decrease physician burnout.

**Table 4:** Study results affinity matrix.

Study result themes or observations	Reference(s)	Frequency, n
Long hours or workload	[25,26,28,33,34,37,38,40,42,45,47]	13
Design issues	[11,30-32,37,44]	7
Poor work-life balance	[25,28,30,34,36,38]	6
EHR^a^ improves quality and safety	[35,41]^b^	3
Loss of autonomy	[25,36]^b^	3
Poor relationships with colleagues	[25,31,36]	3
Cognitive fatigue	[11,31,43]	3
EHR-related or work-related stress	[29,38]	2
Efficiency gains with training	[41]^b^	2
EHR improves clinical workflow	[35,41]	2
Longer on the job	[24,45]	2
System speed or reliability issues	[11,41]	2
EHR improves accuracy of documentation	[41]	1
EHR improves readability	[41]	1
Women more likely to report suicidal ideations	[28]	1
High-quality hospital has high expectations	[24]	1
Alert fatigue	[43]	1
Community-affiliated residents more likely to report suicidal ideations	[28]	1
Comorbidities	[24]	1
EHR improves door-to-doctor and time to decision	[35]	1
Women at a higher risk of burnout	[28]	1
Few paid vacations	[24]	1
Lack of interoperability	[30]	1
Low frequency of exercise	[24]	1
Message complexity	[31]	1
Message content	[31]	1
More night shifts	[24]	1
No time for themselves	[25]	1
Radiologists more likely to report symptoms of burnout	[27]	1
Scribes or physician partners can decrease symptoms of burnout	[46]	1
Seated position causes problems with back or wrist pain and posture	[30]	1
Time spent in EHR affects patient satisfaction	[39]	1

^a^EHR: electronic health record.

^b^Multiple occurrences observed in one study.

### Medical Outcomes Identified With the EHR and Physician Burnout

Table 5 summarizes the medical outcomes observed: 3 themes and 4 individual observations were identified by the reviewers for a total of 10 occurrences in the literature. Of the 25 articles, 20 (80%) did not report medical outcomes. Back pain [30,38], physical fatigue [11,43], and cognitive weariness [11,43] were each mentioned 2 times out of 10 observations (60%). The other medical outcomes were eye strain, work stress, hand or wrist pain, and posture [29,30,38].

**Table 5:** Medical outcomes identified with the electronic health record (EHR) and physician burnout.

Medical outcome theme or observation	Reference(s)	Frequency, n
Back pain	[30,38]	2
Physical fatigue	[11,43]	2
Cognitive weariness	[11,43]	2
Eye strain	[38]	1
Work stress	[29]	1
Hand or wrist pain	[38]	1
Posture	[30]	1
None reported	[24-28,31-37,39-42,44-47]	20

### Patient Satisfaction Impact of EHR

This section is not entirely logical. When we designed this study, we assumed we would find more experiments. We expected to find experiments with and without the presence of the EHR or experiments with control groups to objectively measure interventions to improve physician burnout incident to the EHR. The results of the study searches did not identify any true experiments. There were only 2 pre-post studies. The only experiments identified used training or scribes to help improve physician burnout. Table 6 identifies these as well as all mentions of patient satisfaction in the group of articles analyzed.

Although patients did not prefer a scribe in the room during an exam, their presence did not negatively affect patient satisfaction in a statistically significant manner [40,46]. Only 2 other articles mentioned patient satisfaction. One article mentioned that time in the EHR negatively affects patient satisfaction, and this negatively affects both symptoms of physician burnout and the doctor-patient relationship [24]. The other article identified the time of day the physician is in the EHR during clinic time has a greater effect on patient satisfaction than the amount of time spent with patients [39].

**Table 6:** Patient satisfaction impact of the electronic health record (EHR) and efforts to improve physician burnout.

Patient satisfaction theme or observation	Reference(s)	Frequency, n
Patient satisfaction not affected by scribe or physician partner in clinic during exam	[40,46]	2
EHR time in clinic negatively affects patient satisfaction	[24]	1
Time of day affects patient satisfaction more than time spent with patient	[39]	1
Patient dissatisfaction negatively affects physician burnout	[24]	1
Patient dissatisfaction negatively affects doctor-patient relationship	[24]	1
Not reported	[11,25-38,41-45,47]	21

### Barriers Identified With the EHR and Physician Burnout

Table 7 summarizes the barriers incident to using the EHR to mitigate symptoms of physician burnout. The reviewers identified 8 themes and 8 individual observations, for a total of 56 occurrences in the literature; 2 articles did not identify barriers [24,45].

The theme of “EHR must undergo a redesign” occurred in 12 of 58 occurrences (21%) [11,30,32,35-38,43,44,47]. Researchers echoed their participants’ pleas to improve the design of the EHR; to reduce task repetition, screen clutter, number of clicks per task, and inefficient interfaces; improve the workflow; and reduce unnecessary searching and inefficient data entry. The inefficiencies take time away from patients and make the day longer for the provider, which impacts work-life balance. The inefficiencies lead to “excessive hours spent in the EHR, which exacerbate symptoms of physician burnout.” This theme occurred in 8 of 58 occurrences (14%) [25,26,28,30,34,39,40,44]. The administrative overhead associated with the EHR creates inefficiencies in the standard workflow of seeing patients. This theme occurred also occurred in 8 of 58 occurrences (14%) [31,33,35,38,43,44,47]. Examples of inefficiencies were excessive data entry, illogical workflow, high number of clicks per task, and multiple screens. These inefficiencies lead to excessive hours spent in the EHR, which adversely affects work-life balance and adds to daily frustration levels. This theme occurred in 6 of 58 occurrences (11%) [25,29-31,36,47]. To add to the inefficiencies, providers noted a level of frustration at the speed and reliability issues associated with the EHR [32,37,38,44]. Participants noted communication technologies and data-sharing processes that are cumbersome and counterproductive, unpredictable system response times, and lack of hardware and infrastructure to make the EHR faster and more reliable. On the topic of administrative time in the EHR, participants noted that administrative time in the EHR takes time away from the clinic and patients. This theme occurred 4 out of 58 occurrences (7%) [25,29,30,42]. Some of the inefficiencies highlighted by providers were that some patient information is not available due to lack of interoperability. This theme occurred in 3 of 58 occurrences (5%) [32,38,44]. This lack of availability creates data overload, which complicates data integration efforts. It often prevents linking to legacy systems, and it creates barriers with data sharing between organizations. Inefficiencies like number of clicks per process encumber efficient workflows. This theme also occurred in 3 of 58 occurrences (5%) [11,30,35]. As mentioned in the table for general results, 2 studies noted that EHR users felt a loss of autonomy [25,36]. Other observations only occurred once in the literature [27,36,40,41,44,46]. One study noted that the EHR does not coordinate care [27]. A study that used scribes or physician partners to enter data into the EHR during the exam noted that patients do not like this practice [40]. Another study that used scribes in the exam room noted the cost to the organization for this practice [40]. A study that used training to improve provider efficiency noted this training takes time away from the clinic [41]. One study noted a lack of support by the organization for EHR tools and efficiency [36]. Another study noted that the EHR does not have a standard vocabulary [44].

**Table 7:** Barriers to the electronic health record (EHR) and physician burnout.

Barrier theme or observation	Reference(s)	Frequency, n
EHR must undergo redesign	[11,30,32,35-38,43,44,47]^a^	12
Excessive hours spent in the EHR exacerbate symptoms of physician burnout	[25,26,28,30,34,39,40,44]	8
The administrative overhead of the EHR is not conducive to efficient workflow	[31,33,35,38,43,44,47]^a^	8
Excessive hours spent in the EHR affect work-life balance	[25,29-31,36,47]	6
EHR reliability and speed	[32,37,38,44]	4
Administrative time in the EHR takes time away from clinic and patients	[25,29,30,42]	4
Some patient information is not available due to lack of interoperability	[32,38,44]	3
High number of clicks per process is inefficient	[11,30,35]	3
EHR creates a loss of autonomy	[25,36]	2
EHR does not help coordinate care	[27]	1
Some patients do not like scribes or physician partners in the exam room	[40]	1
EHR training takes time away from clinic	[41]	1
Scribes or physician partners cost more money	[46]	1
Lack of supporting practice environment	[36]	1
No standardized vocabulary	[44]	1
EHR investment inhibits short-term profit	[44]	1
Not reported	[24,45]	2

^a^Multiple occurrences observed in one study.

### Facilitators Identified With the EHR and Physician Burnout

Table 8 summarizes the facilitators incident to using the EHR to mitigate symptoms of physician burnout: 6 themes and 12 individual observations were identified by the reviewers for a total of 27 occurrences in the literature. Facilitators were not identified in 11 articles [11,26,27,29,30,34,36,37,42,43,47].

The theme of “presence of a scribe or physician partner relieves symptoms of burnout” occurred in 3 of 27 occurrences (11%) [38,40,46]. Although this practice incurs a cost to the organization, the use of either a scribe or a physician partner to enter data into the EHR during the encounter enables the physician to focus on the patient rather than negotiating the EHR, and the scribe’s time entering data into the EHR can easily be offset by a savings in provider administrative time later. This practice decreases appointment time and enables the provider to work a standard day instead of spending so much time after clinic hours catching up with the administrative side of the day’s encounters. Geriatrics practices that leveraged scribes in this manner experienced an average of 4 minutes less per encounter for an average of 48 minutes per 4-hour session. When the administrative time after the encounter was accounted for, the savings was 88 minutes per 4-hour session. Internal medicine experienced a 2 minute per patient savings for a total of 92 minutes per 4-hour session, counting administrative time saved. Another set of studies found training would increase physician efficiency in the EHR. This theme also occurred in 3 of 27 occurrences (11%) [32,38,41]. Training decreased their frustration with the system and shortened their work day. This practice improved work-life balance and decreased symptoms of burnout. A similar theme found localized workflow redesign relieves symptoms of burnout. This theme also occurred in 3 of 27 occurrences (11%) [31,39,40]. The most common workflow redesign was preparation for encounters, which also increased patient satisfaction. Similar to training and workflow redesign, it was discovered that customized templates also increased efficiencies. This theme occurred in 2 of 27 occurrences (7%) [31,33]. This practice also increased accuracy and completeness of documentation [44], which increases quality of care. The theme “small group sessions” also occurred in 2 of 27 occurrences (7%) [28]. This theme focused on development of young providers. This development focused on emotional and professional development. These sessions also helped establish rapport among providers. Two studies highlighted how the EHR increases safety [35,44]. The readability of orders and intelligence built into the system to alert when doses are outside of a standard range increase safety and decrease admission decision time and length of stay [35]. The other observations were only identified once [11,24,25,28-32,34-38,41,43-45,47]. One study mentioned that, although improving the EHR will help with the burden of care, it also is important to schedule regular exercise to help providers cope with the stress of care [24]. One study highlighted how a focus on the mission of care, rather than the administration of the encounter, decreases symptoms of burnout [25]. One study highlighted the ability of the EHR to rapidly access patient data, which saves the provider time searching through a paper record [44]. Based on the conclusions of other studies, it is the process of finding this information that is key. One study highlighted the importance of provider experience (years as a provider and years in the EHR) to appreciate the usefulness of this tool [45]. Institutional endorsement of the EHR is also important [41]. This is important because it increases user acceptance of the system. A study in China found that providers who take their annual vacation tended to report fewer symptoms of burnout [24]. A study in Tehran identified the capability for the EHR to enable computerized analysis; however, this capability should be found easily rather than taking time to hunt for the feature [44]. Through training programs and customization, the EHR can increase the speed of delivery of care [44].

**Table 8:** Facilitators to the electronic health record (EHR) and physician burnout.

Facilitator theme or observation	Reference(s)	Frequency, n
Presence of scribe or physician partner relieves symptoms of burnout	[38,40,46]	3
Training increases efficiency	[32,38,41]	3
Localized workflow redesign relieves symptoms of burnout	[31,39,40]	3
Local customization (eg, templates, menus) improves efficiency	[31,33]	2
Small group sessions	[28]^a^	2
EHR increases safety	[35,44]	2
Exercise relieves symptoms of burnout	[24]	1
Focus on mission of care relieves symptoms of burnout	[25]	1
EHR enables rapid access to information	[44]	1
Level of physician experience with EHR increases perceived usefulness of EHR	[45]	1
Institutional endorsement of EHR increases user acceptance of EHR	[41]	1
Annual vacation relieves symptoms of burnout	[24]	1
EHR decreases admission decision time	[35]	1
EHR decreases length of stay	[35]	1
EHR decreases duplicate testing	[44]	1
EHR increases speed of delivery of care	[44]	1
EHR increases accuracy of documentation	[44]	1
EHR enables computerized analysis and interpretation of data	[44]	1
Not reported	[11,26,27,29,30,34,36,37,42,43,47]	11

^a^Multiple occurrences observed in one study.

## Discussion

### Summary of Evidence

The preponderance of evidence supports the claim that the EHR needs an overall redesign to increase efficiency of providers. However, very few empirical studies published in the studied years could be found to measure the deficiencies. One study measured pupillometry, one measured cognitive load, and another measured cognitive weariness [11,31,43], but claims of inefficiencies were largely the result of surveys. Clearly, providers spend a great deal of time in the EHR managing the administrative necessities of the system; however, studies with training and local customization of templates and workflow greatly improved efficiencies and decreased symptoms of burnout [33,41]. The creative use of scribes and physician partners to relieve providers of some of the real-time documentation burden showed statistically significant improvement in burnout symptoms, but they come at a price of an increased cost to employ them and a slight decrease in patient satisfaction (not statistically significant) [40,46].

From the practitioners’ points of view, they wanted to know what factors remain in their sphere of influence to assuage the effects of physician burnout. Factors associated with the EHR cited in relation to physician burnout were usability, workflow, and documentation time [8-13]. Workflow can be redesigned and customized to the user, and documentation can be performed with the use of scribes or physician partners [40,46]. The remaining factor was usability, which can only be managed in a large redesign effort. Practitioners should focus on robust and ongoing training, customization of local templates, and workflow redesign. They should weigh the economics of scribes or physician partners against the decrease in symptoms of burnout. If increasing the prevalence of symptoms of burnout increases physician turnover [2-4], certainly reducing symptoms of burnout will decrease turnover. Some best practices identified in the literature to reduce burnout were taking annual vacation [24], focusing the organization on the mission of care rather than the administration of it [25], scheduling small group sessions to help emotionally equip young providers [28], institutional endorsement of the EHR [41], and the use of regular exercise to manage stress [24]. However, these techniques do not improve the usability of the EHR, but they were identified as practices to decrease the symptoms of burnout.

Future research should empirically measure the redesign factor of usability. What aspects of usability can be improved? Are navigation issues in the EHR specific to each vendor? Are there best practices from one vendor that can be applied to other vendors without infringing upon proprietary secrets? What mental processes in the physician workflow can be directly mapped into the menus of the EHR?

### Limitations

A limitation to this review is the selection of 5 years. It was originally assumed there would be a plethora of studies on the topic of physician burnout incident to the EHR, but we found a dearth of empirical studies on the topic. There were plenty of opinion articles but very little empirical evidence. This review could have been improved by expanding the time period to 10 years, but technology advances rapidly, and reconciling the observations over a decade might have been counterproductive.

### Conclusion

Although physician burnout incident to the EHR has been documented, several best practices exist to overcome 2 of the 3 factors associated with the EHR: workload and documentation time. The effect of these factors can be assuaged through workload redesign, customized templates, training, and the use of physician partners or scribes in the exam room. The third factor of usability can only be overcome through a redesign of the EHR. Practitioners should focus on the former factors, which are within their sphere of control. EHR vendors should organize empirical studies to identify targeted areas of improvement to optimize the usability of the system.

## Appendix

Multimedia Appendix 1 Observation-to-theme conversion for results and medical outcomes.

Multimedia Appendix 2 Observation-to-theme conversion for patient satisfaction, barriers, and facilitators.

Multimedia Appendix 3 Other observations incident to review.

## Abbreviations

EHR: electronic health record
JHNEBP: Johns Hopkins Nursing tool for Evidence Based Practice
PICOS: participants, intervention, comparison of results to control, outcome (medical), study design
PRISMA: Preferred Reporting Items for Systematic Reviews and Meta Analyses
