# Supplementary material for: Physician Burnout and the Electronic Health Record Leading Up to and During the First Year of COVID-19: Systematic Review
Source: J Med Internet Res. 2022 Mar 31;24(3):e36200. doi: 10.2196/36200 (PMC9015762; doi:10.2196/36200)
Supplement: Multimedia Appendix 1 [file jmir_v24i3e36200_app1.docx]

**Multimedia Appendix 1.** Observation-to-theme conversion for results and medical outcomes.

| Authors | Results (compared with control group) | Result themes | Medical outcomes reported | Medical outcome themes |
| --- | --- | --- | --- | --- |
| Hu et al [24] | No control group. Low frequency of exercise | Low frequency of exercise | none reported | None reported |
|  | having comorbidities contributes to burnout | Comorbidities |  |  |
|  | working in a high-quality hospital contributes to burnout | High-quality hospital has high expectations |  |  |
|  | more night shifts contribute to burnout | More night shifts |  |  |
|  | more years of work experience contributes to burnout | Longer on the job |  |  |
|  | Fewer paid vacation days contributes to burnout | Few paid vacations |  |  |
| Rialon et al [25] | No control group. Reasons for burnout: over half respondents work 60-80 hours per week, | Long hours / workload | none reported | None reported |
|  | 6% felt they had time for themselves, | No time for themselves |  |  |
|  | work-life balance | Poor work-life balance |  |  |
|  | most experience loss of autonomy, | Loss of autonomy |  |  |
|  | poor relationships with colleagues | Poor relationships with colleagues |  |  |
|  | administrative and clinical burdens. | Long hours / workload |  |  |
| Giess et al [27] | No control group. Radiologists more likely to report burnout than non-radiologists (not statistically significant) | Radiologists more likely to report symptoms of burnout | none reported | None reported |
| Kinslow et al [28] | No control group. Residents reporting training at community teaching hospital programs |  | none reported | None reported |
|  | female gender is at higher risk for reporting burnout. | Females at a higher risk for burnout |  |  |
|  | work-life balance | Poor work-life balance |  |  |
|  | increased number of hours worked per week are at higher risk for reporting burnout. | Long hours / workload |  |  |
|  | Females were at increased risk of reporting suicidal ideation | Females more likely to report suicidal ideations |  |  |
|  | community-affiliated residents were at increased risk of reporting suicidal ideation, | Community-affiliated residents more likely to report suicidal ideations |  |  |
| Anderson et al [26] | No control group. family medicine trainees and faculty saw between one and 164 patients monthly, spent between 17 and 217 minutes in the EHR per patient, and spent between 0 and 33 hours in the EHR after hours per month. | Long hours / workload | none reported | None reported |
| Khairat et al [11] | No control group. Physician fatigue as measured by pupillometry | Cognitive fatigue | Fatigue | Physical fatigue |
|  | EHR efficiency | Design issues | Mental fatigue | Cognitive weariness |
| Murphy et al [31] | No control group. Primary complaints were message processing complexity, inbox interface design, cognitive load, team communication, and inbox message content | Message complexity | none reported | None reported |
|  | Interface design | Design issues |  |  |
|  | Cognitive load | Cognitive fatigue |  |  |
|  | Team communication | Poor relationships with colleagues |  |  |
|  | Inbox message content | Message content |  |  |
| Tran et al [34] | No control group. 39% (41) reported burnout based on clinical time and patient workloads. More than half of the providers with greater than 0.9 clinical FTE reported burnout while only 33% with less than 0.6 clinical FTE reported burnout. | Long hours / workload | none reported | None reported |
|  | work-life balance | Poor work-life balance |  |  |
| Gardner et al [29] | No control group. 26% reported burnout. 70% reported HIT-related stress, with the highest prevalence in primary care-oriented specialties. | EHR-related / work related stress | Work-related stress | Work stress |
|  |  |  |  |  |
| Kroth et al [30] | No control group. The EHR design and use factors identified by clinicians as most associated with stress and burnout were excessive data entry, | Design issues | Problems with posture, | Posture |
|  | long cut-and-pasted notes, | Design issues | back pain | Back pain |
|  | interoperability, | Lack of interoperability |  |  |
|  | interference with work-life balance, | Poor work-life balance |  |  |
|  | problems with posture, and pain. | Seated position causes problems with back/wrist pain and posture |  |  |
| Sieja et al [33] | Symptoms of burnout decreased post implementation by 5% | Long hours / workload | none reported | None reported |
| Quinn et al [32] | No control group. Existing communication and data sharing processes are inefficient. The EHR needs design improvement. | Design issues | none reported | None reported |
|  |  |  |  |  |
|  |  |  |  |  |
| Robinson and Kersey [41] | No control group. Most physicians (85%–98% across all programs) reported improved quality, readability, and clinical accuracy of documentation; fewer medical errors; and increased efficiency in chart review and data retrieval due to the training. | EHR improves quality / safety | none reported | None reported |
|  | Improved readability | EHR improves readability |  |  |
|  | Improved clinical accuracy of documentation | EHR improves clinical workflow |  |  |
|  | Fewer medical errors | EHR improves quality / safety |  |  |
|  | Improved documentation | EHR improves accuracy of documentation |  |  |
|  | Increased efficiency of chart review | Efficiency gains with training |  |  |
|  | Improved data retrieval | System speed / reliability issues |  |  |
|  | Overall time savings: 78% estimated a time savings of 4 to 5 minutes or more per hour. | Efficiency gains with training |  |  |
| Pozdnyakova et al [40] | Pre-pilot, all six physicians agreed that they felt rushed during clinic; all disagreed with this statement post-pilot. Only two (33%) were satisfied with clinic workflow pre-pilot, but all physicians were satisfied post-pilot. Before the pilot, five physicians (83%) agreed that “too much time in clinic is spent working on the computer,” whereas post-pilot, no physicians agreed. Only one physician reported burnout symptoms at baseline, which did not change post-pilot. | Long hours / workload | none reported | None reported |
|  |  |  |  |  |
| Marmor et al [39] | No control group. During the daytime hours, an inverse relationship occurred for time spent with patient and the following domains: provider showed patient respect, provider knew patient's history, overall communication quality, and likelihood to recommend provider | Time spent in EHR affects patient satisfaction | none reported | None reported |
| Denton et al [35] | No control group. EHRs improved their clinical workflow, | EHR improves clinical workflow | none reported | None reported |
|  | especially on MU-related activities including door-to-doctor time and admit decision time. | EHR improves door-to-doctor and time to decision |  |  |
|  | EHR use also affected physicians work efficiency, quality of care provided, and overall patient safety. | EHR improves quality / safety |  |  |
| Kroth et al [38] | No control group. Only 22% indicated sufficient time for documentation, | Long hours / workload | Eye strain | Eye strain |
|  | 56% noted “a great deal of stress” because of their job, | EHR-related / work related stress | hand/wrist pain | Hand/wrist pain |
|  | 42% reported “poor” or “marginal” control over workload. | Long hours / workload | back pain | Back pain |
|  | 42% reported “poor” or “marginal” control over workload. Even though 90% reported EHR proficiency, 56% indicated EHR time at home was “excessive” or “moderately high.” | Poor work-life balance |  |  |
|  |  |  |  |  |
| Hauer et al [36] | No control group. Primary causes of physician burnout include utilization and interactions with electronic health records (EHR), | Loss of autonomy | none reported | None reported |
|  | lack of a supportive practice environment, | Poor relationships with colleagues |  |  |
|  | the loss of autonomy, | Loss of autonomy |  |  |
|  | and poor work/life balance. | Poor work-life balance |  |  |
| Young et al [42] | No control group. The mean (SD) visit length was 35.8 (16.6) minutes, not counting resident precepting time. The mean time components included 2.9 (3.8) minutes working in the EHR prior to entering the room, 16.5 (9.2) minutes of face-to-face time not working in the EHR, 2.0 (2.1) minutes working in the EHR in the room (which occurred in 73.4% of the visits), 7.5 (7.5) minutes of non-face time (mostly EHR time), and 6.9 (7.6) minutes of EHR work outside of normal clinic operational hours (which occurred in 64.6% of the visits). The total time and total EHR time varied only slightly between faculty physicians, third-year and second-year residents | Long hours / workload | none reported | None reported |
| Khairat et al [37] | No control group. Highest frustrations were: remembering menu/button names and commands, | Design issues | none reported | None reported |
|  | Tasks not intuitive | Long hours / workload |  |  |
|  | system speed and reliability | System speed / reliability issues |  |  |
| Arndt et al [47] | No control group. Time and motion study in face-to-face clinic over 6 weeks. Clinicians spent 355 minutes (5.9 hours) of an 11.4-hour workday in the EHR per weekday per 1.0 clinical full-time equivalent: 269 minutes (4.5 hours) during clinic hours and 86 minutes (1.4 hours) after clinic hours. Clerical and administrative tasks including documentation, order entry, billing and coding, and system security accounted for nearly one-half of the total EHR time (157 minutes, 44.2%). Inbox management accounted for another 85 minutes (23.7%). | Long hours / workload | none reported | None reported |
|  |  |  |  |  |
|  |  |  |  |  |
|  |  |  |  |  |
| Shahmoradi et al [44] | No control group. Users appreciate the overall benefit of the EHR, but it creates more work for the provider and could use improvements of design. | Design issues | none reported | None reported |
| Gregory et al [43] | No control group. Alert workload was related to two of the three dimensions of burnout, including physical fatigue (p = 0.02) and, when controlling for organizational tenure. | Alert fatigue | Physical fatigue | Physical fatigue |
|  | cognitive weariness (p = 0.04) | Cognitive fatigue | Cognitive weariness | Cognitive weariness |
| Jamoom et al [45] | No control group. 70% physicians attribute administrative burden to the EHR. | Long hours / workload | none reported | None reported |
|  | Physicians with more EHR experience attributed more positive aspects to the EHR. | Longer on the job |  |  |
| Reuben et al [46] | No control group. 97 % indicated that they placed great value on working with the Physician Partners, and 70 % indicated that they would be willing to add two patients per session to their schedule to do so. | Scribes / physician partners can decrease symptoms of burnout | none reported | None reported |
