# Supplementary material for: Physician Burnout and the Electronic Health Record Leading Up to and During the First Year of COVID-19: Systematic Review
Source: J Med Internet Res. 2022 Mar 31;24(3):e36200. doi: 10.2196/36200 (PMC9015762; doi:10.2196/36200)
Supplement: Multimedia Appendix 2 [file jmir_v24i3e36200_app2.docx]

**Multimedia Appendix 2.** Observation-to-theme conversion for patient satisfaction, barriers, and facilitators.

| Authors | Patient satisfaction | Patient satisfaction themes | Barriers | Barrier themes | Facilitators | Facilitator themes |  |
| --- | --- | --- | --- | --- | --- | --- | --- |
| Hu et al [24] | Patients seem to be unsatisfied with the services they received, which contributes to a poor doctor-patient relationship and an increase in burnout among doctors and nurses. | EHR time in clinic negatively affects patient satisfaction | not reported | Not reported | Exercise could help relieve symptoms of burnout. | Exercise relieves symptoms of burnout |  |
|  | Patient dissatisfaction negatively affects doctor-patient relationship | Patient dissatisfaction negatively affects doctor-patient relationship |  |  | Annual vacation could also help. | Annual vacation relieves symptoms of burnout |  |
|  | Patient dissatisfaction negatively affects physician burnout | Patient dissatisfaction negatively affects physician burnout |  |  |  |  |  |
|  |  |  |  |  |  |  |  |
| Rialon et al [25] | not reported | Not reported | Excessive hours spent in the EHR detrimentally affect work-life balance | Excessive hours spent in the EHR affects work-life balance | Institutional and departmental efforts to facilitate dedicated time for the components of the academic mission that physicians find personally meaningful | Focus on mission of care relieves symptoms of burnout |  |
|  |  |  | Time spent in the EHR contributes to physician burnout | Excessive hours spent in the EHR exacerbates symptoms of physician burnout |  |  |  |
|  |  |  |  |  |  |  |  |
|  |  |  | Documentation takes time away from patients | Administrative time in the EHR takes time away from clinic and patients |  |  |  |
|  |  |  |  |  |  |  |  |
|  |  |  |  |  |  |  |  |
| Giess et al [27] | not reported | Not reported | Negative correlation of time spent in the EHR and coordinating care efficiently | EHR does not help coordinate care | not reported | Not reported |  |
| Kinslow et al [28] | not reported | Not reported | Long hours spent interfacing with EHR contribute to symptoms of burnout | Excessive hours spent in the EHR exacerbates symptoms of physician burnout | Targeted small group sessions, early emphasis on emotional and professional development may help alleviate the multiple factors that contribute to burnout symptomatology. | Small group sessions |  |
|  |  |  |  |  | Facilitation of rapport building amongst residents may help alleviate the multiple factors that contribute to burnout symptomatology. | Small group sessions |  |
| Anderson et al [26] | not reported | Not reported | Family medicine residents spend a significant amount of time completing EHR tasks after hours. | Excessive hours spent in the EHR exacerbates symptoms of physician burnout | not reported | Not reported |  |
| Khairat et al [11] | not reported | Not reported | Design causes unnecessary searching and data entry | EHR must undergo redesign | not reported | Not reported |  |
|  |  |  | Design causes inefficiencies | High number of clicks per process is inefficient |  |  |  |
| Murphy et al [31] | not reported | Not reported | High number of clicks required per task (design), workload, workflow | The administrative overhead of the EHR is not conducive to efficient workflow | Locally designed templates will improve quality and workflow | Local customization (templates, menus, etc.) improves efficiency |  |
|  |  |  | Workload | Excessive hours spent in the EHR affects work-life balance | user-customized interface may improve workflow (but complicates support) | Localized workflow redesign relieves symptoms of burnout |  |
|  |  |  | Workflow | The administrative overhead of the EHR is not conducive to efficient workflow |  |  |  |
|  |  |  |  |  |  |  |  |
|  |  |  |  |  |  |  |  |
| Tran et al [34] | not reported | Not reported | Workload is highly associated with self-reported burnout. | Excessive hours spent in the EHR exacerbates symptoms of physician burnout | not reported | Not reported |  |
|  |  |  |  |  |  |  |  |
| Gardner et al [29] | not reported | Not reported | Poor marginal documentation time, | Administrative time in the EHR takes time away from clinic and patients | not reported | Not reported |  |
|  |  |  | high EHR time at home, EHR adds to daily frustration. | Excessive hours spent in the EHR affects work-life balance |  |  |  |
| Kroth et al [30] | not reported | Not reported | EHR design (multiple windows, task repetition, workflow (efficiency, effective quality of care), work-life balance | EHR must undergo redesign | not reported | Not reported |  |
|  |  |  | EHR is time consuming, | Excessive hours spent in the EHR exacerbates symptoms of physician burnout |  |  |  |
|  |  |  | too many clicks | High number of clicks per process is inefficient |  |  |  |
|  |  |  | workflow | Administrative time in the EHR takes time away from clinic and patients |  |  |  |
|  |  |  | work-life balance | Excessive hours spent in the EHR affects work-life balance |  |  |  |
| Sieja et al [33] | not reported | Not reported | EHR design, workflow | The administrative overhead of the EHR is not conducive to efficient workflow | localized tool development can decrease symptoms of physician burnout | Local customization (templates, menus, etc.) improves efficiency |  |
| Quinn et al [32] | not reported | Not reported | Communication technologies and data sharing processes are cumbersome and counterproductive. | EHR reliability and speed | Training increases EHR proficiency. | Training increases efficiency |  |
|  |  |  | The EHR creates data overload and fragmentation which complicates data integration efforts. | Some patient information is not available due to lack of interoperability |  |  |  |
|  |  |  | Design needs improving. | EHR must undergo redesign |  |  |  |
| Robinson and Kersey [41] | not reported | Not reported | Time for training | EHR training takes time away from clinic | Institutional endorsement | Institutional endorsement of EHR increases user acceptance of EHR |  |
|  |  |  |  |  | Training increases EHR proficiency. | Training increases efficiency |  |
| Pozdnyakova et al [40] | Patient satisfaction was not different between patients who had clinic visits with vs. without scribe overall or by age, gender, and race. Compared to patients 65 years or older, younger patients were more likely to report that the physician was more attentive and provided more education during visits with the scribe present (p = 0.03 and 0.02, respectively). Male patients were more likely to report that they disliked having a scribe (p = 0.03). | Patient satisfaction not affected by scribe / physician partner in clinic during exam | Presence of a scribe during medical encounters negatively affects patient satisfaction in some cases | Some patients do not like scribes / physician partners in the exam room | Presence of a scribe during medical encounters positively affects symptoms of physician burnout in all cases. | Presence of scribe / physician partner relieves symptoms of burnout |  |
|  |  |  |  | Excessive hours spent in the EHR exacerbates symptoms of physician burnout |  | Localized workflow redesign relieves symptoms of burnout |  |
| Marmor et al [39] | During the daytime hours, an inverse relationship occurred for time spent with patient and the following domains: provider showed patient respect, provider knew patient's history, overall communication quality, and likelihood to recommend provider. This relationship did not occur after hours. | Time of day affects patient satisfaction more than time spent with patient | After hours documentation can detrimentally affects physician satisfaction and contribute to physician burnout. | Excessive hours spent in the EHR exacerbates symptoms of physician burnout | Proper work process design helps physicians prepare for encounters with patients and decreases after hours documentation time, which increases patient satisfaction. | Localized workflow redesign relieves symptoms of burnout |  |
| Denton et al [35] | not reported | Not reported | EHR design (multiple windows, task repetition, screen clutter, number of clicks) | EHR must undergo redesign | EHR increases safety, decreases admission decision time | EHR increases safety |  |
|  |  |  | High number of clicks required per task (design) | High number of clicks per process is inefficient | EHR decreases admission decision time | EHR decreases admission decision time |  |
|  |  |  | workflow (efficiency, effective quality of care) | The administrative overhead of the EHR is not conducive to efficient workflow | EHR decreases length of stay | EHR decreases length of stay |  |
| Kroth et al [38] | not reported | Not reported | Focus group themes included HICT “successes” where all patients’ information is accessible from multiple locations. | EHR must undergo redesign | Additional EHR training would increase proficiency, | Training increases efficiency |  |
|  |  |  | HICT “stressors” included inefficient user interfaces, | EHR must undergo redesign | an increase in organizational scribes would decrease the amount of time physicians spend in the EHR. | Presence of scribe / physician partner relieves symptoms of burnout |  |
|  |  |  | unpredictable system response times, | EHR reliability and speed |  |  |  |
|  |  |  | poor interoperability between systems and | Some patient information is not available due to lack of interoperability |  |  |  |
|  |  |  | excessive data entry. | The administrative overhead of the EHR is not conducive to efficient workflow |  |  |  |
| Hauer et al [36] | not reported | Not reported | Poor interface of EHR (design), | EHR must undergo redesign | not reported | Not reported |  |
|  |  |  | lack of supporting practice environment, | Lack of supporting practice environment |  |  |  |
|  |  |  | loss of autonomy, | EHR creates a loss of autonomy |  |  |  |
|  |  |  | poor work/life balance. | Excessive hours spent in the EHR affects work-life balance |  |  |  |
| Young et al [42] | not reported | Not reported | Time spent working in the EHR versus working face-to-face with the patient. | Administrative time in the EHR takes time away from clinic and patients | not reported | Not reported |  |
| Khairat et al [37] | not reported | Not reported | EHR design, system speed, system reliability | EHR must undergo redesign | not reported | Not reported |  |
|  |  |  | Reliability and speed | EHR reliability and speed |  |  |  |
|  |  |  |  |  |  |  |  |
| Arndt et al [47] | not reported | Not reported | Time spent working in the EHR versus working face-to-face with the patient and work-life balance. Design issues. Workload. Work processes. | EHR must undergo redesign | not reported | Not reported |  |
|  |  |  | Design issues | EHR must undergo redesign |  |  |  |
|  |  |  | work-life balance | Excessive hours spent in the EHR affects work-life balance |  |  |  |
|  |  |  | Workflow | The administrative overhead of the EHR is not conducive to efficient workflow |  |  |  |
| Shahmoradi et al [44] | not reported | Not reported | Lack of hardware and infrastructure, | EHR reliability and speed | EHR enables timely and rapid access to information, | EHR enables rapid access to information |  |
|  |  |  | EHR is time consuming, | Excessive hours spent in the EHR exacerbates symptoms of physician burnout | reduces duplicate testing, | EHR decreases duplicate testing |  |
|  |  |  | cannot link to legacy system of records, | Some patient information is not available due to lack of interoperability | increases speed of delivery, | EHR increases speed of delivery of care |  |
|  |  |  | increases workload of physicians, | The administrative overhead of the EHR is not conducive to efficient workflow | accurate record of encounters, | EHR increases accuracy of documentation |  |
|  |  |  | inhibits short-term profit, | EHR investment inhibits short-term profit | prevents medical error, | EHR increases safety |  |
|  |  |  | programming shortfalls (design), | EHR must undergo redesign | enables computerized analysis and interpretation. | EHR enables computerized analysis and interpretation of data |  |
|  |  |  | no standardized vocabulary. | No standardized vocabulary |  |  |  |
| Gregory et al [43] | not reported | Not reported | EHR design | EHR must undergo redesign | not reported | Not reported |  |
|  |  |  | workflow | The administrative overhead of the EHR is not conducive to efficient workflow |  |  |  |
| Jamoom et al [45] | not reported | Not reported | not reported | not reported | Experience positively affects perceived usefulness of EHR | Level of physician experience with EHR increases perceived usefulness of EHR |  |
|  |  |  |  |  |  |  |  |
| Reuben et al [46] | Of the 125 patients surveyed, 93 % responded “no” when asked whether they felt uncomfortable having a Physician Partner in the room, and 86 % of patients felt that the Physician Partners helped their visits run smoothly. | Patient satisfaction not affected by scribe / physician partner in clinic during exam | Cost of physician partners | Scribes or physician partners cost more money | Physician partners decreases appointment time, decreases symptoms of physician burnout, and could enable expansion of scheduling (additional patients). Presence of physician partners showed no negative effect on patient satisfaction. | Presence of scribe / physician partner relieves symptoms of burnout |  |
