# Supplementary material for: Physician Burnout and the Electronic Health Record Leading Up to and During the First Year of COVID-19: Systematic Review
Source: J Med Internet Res. 2022 Mar 31;24(3):e36200. doi: 10.2196/36200 (PMC9015762; doi:10.2196/36200)
Supplement: Multimedia Appendix 3 [file jmir_v24i3e36200_app3.docx]

**Multimedia Appendix 3.** Other observations incident to review.

| Authors | Sample size | Bias within study | Effect size | Country of origin | Statistics used | Strength of evidence | Quality of evidence |
| --- | --- | --- | --- | --- | --- | --- | --- |
| Hu et al [24] | 2411 | Most participants were young (Under 40) | not reported | China | Nonparametric tests and chi-square tests | III | B |
|  |  |  |  |  |  |  |  |
| Rialon et al [25] | 413 | Mainly seasoned white males. Low survey response rate which may not be representative of the entire membership of APSA, Unable to associate stress between race. | not reported | United States | Descriptive, Chi square test | III | B |
| Giess et al [27] | 1494 | Single academic institution, radiology chair encouraged participation. Could not analyze differences between individual nonradiology specialties and radiology. Survey did not address differences in types of clinical duties which might affect burnout. | not reported | United States | Whitney-Mann U, Chi square analysis | III | B |
| Kinslow et al [28] | 81 | Recall and selection bias. Respondents experiencing higher burnout rates were more inclined to complete the survey, as well as more inclined to report contributing factors. Timing of distribution caused small sample size due to COVID-19 impact. | not reported | United States | IBM SPSS Statistics Version 26.0 (Armonk, NY), Chi-square test, multivariable logistic regression, survey was reported in line with the STOCSS criteria | III | B |
| Anderson et al [26] | 24 | Limited ability to measure faculty EHR time and proximity of the study to an EHR transition. Current methods for monitoring time spent in the EHR do not allow to differentiate between time a faculty physician spends precepting a resident’s patient versus a patient encounter completed on their own. The researchers gathered data near an EHR transition, and while they allowed for 3 months of use prior to starting the retrospective collection, hence improving efficiency has likely occurred. | not reported | United States | Descriptive statistics. Data were analyzed using MedCalc statistical software. | III | B |
| Khairat et al [11] | 25 | Localized study | not reported | United States | Summary and descriptive statistics. Pearson correlation coefficients calculated for all outcomes. Analysis conducted on SPSS version 22.0. | II | A |
| Murphy et al [31] | 25 | localized study | not reported | United States | Inductive thematic approach | III | B |
| Tran et al [34] | 107 | small sample size | not reported | United States | A combination of descriptive analytics and simple linear regression. Fisher’s Exact Test was used to determine whether there were statistically significant associations between self-reported burnout and certain categorial characteristics. Simple linear regression estimated the effect of burnout on various performance indicators once controlling for clinical FTE. | III | B |
| Gardner et al [29] | 4197 | The response rate may affect generalizability. Some differences existed between respondents and non-respondents. Generalizability may also be limited by administration in a single state. Second, physicians may have been reluctant to respond with the full extent of their burnout symptoms because the survey was not anonymous and because it was administered by RIDOH, which also oversees medical licensing in the state. Thus, reported prevalence of burnout symptoms and stress may be an underestimate. Third, the survey was administered electronically, which means that physicians who are more comfortable with computers may be more likely to respond. Finally, the study design does not allow to determine the degree to which stresses such as insufficient documentation time are related to technology vs. the burden of the work itself. Comparing the responses of physicians with EHRs to those without EHRs does support an important role for technology, but the relatively low number of physicians in the sample without EHRs limits the interpretation. | not reported | United States | Univariable statistics were generated to describe the sample characteristics and the prevalence of burnout, HIT-related stress, and EHR remote access. The researchers used bivariable chi-square tests (2-sided) to compare age, gender, and specialty among respondents vs. non-respondents. Significance was assessed at the 0.05 level. Multivariable logistic regression was used to measure the association between burnout and each measure of HIT-related stress, while controlling for respondent demographics, practice characteristics, and the other measures of HIT-related stress. Because 2 of the 3 HIT-related stress measures incorporate use of an EHR, the regression analyses included only physicians who reported using an EHR. Sensitivity analysis was performed to examine whether including each of the 3 HIT-related stress measures with its ordinal response categories (vs. dichotomized response categories) would affect any measures’ association with burnout. Using an ordered logit model, the researchers also performed a sensitivity analysis with the dependent variable (burnout) represented by its ordinal response categories, instead of the dichotomized response categories included in the primary analysis. Another sensitivity analysis included vendor type in the regression model, using the 3 most commonly reported vendors (Epic Systems, eClinicalWorks, and Cerner), to examine whether vendor type independently predicted burnout. | III | B |
| Kroth et al [30] | 282 | There was no variety of physicians used in the study. This study has limitations, including its cross-sectional nature and the use of self-reported metrics. One needs to consider response bias, given the 44.1% response rate. The relatively modest sample size limits validity. As respondents came from only 3 institutions, these results may not be more widely generalizable. The mapping of the paper instrument’s Likert scales to the REDCap slider bars scale may have introduced some bias. Despite using validated instruments to measure burnout and stress, the survey relied on the respondents’ own definitions. Self-reported metrics may underrepresent the numbers at risk. All respondents were grouped together for this analysis, which does not account for possible intragroup differences, such as between physicians and advanced practice clinicians. | not reported | United States | Linear and logistic regressions were used to estimate associations of work conditions with stress on a continuous scale and burnout as a binary outcome from an ordered categorical scale. Linear regression was used to determine the association of focus group–identified variables (eg, work conditions, EHR design and use factors, and coping strategies) with clinician-reported stress, which the researchers scored according to the Motowidlo 4-item measure,14 and burnout. β̂ was used to estimate the magnitude and direction of association, and it was calculated using the least-square estimation technique. We used logistic regression with stepwise selection, which is a combination of the forward and backward selection techniques, to estimate the association of focus group–identified variables with the odds of clinician-reported burnout, which we measured as a binary outcome based on a single question (with burnout representing endorsement of any choice with the word burnout in it).14 We used construct variables created to summarize the associations of variables within the same domain with stress and burnout. To develop the final regression model for stress, variables with R2 greater than 0.10 in the univariate analysis or that were determined to be of special interest were considered candidate variables for the multivariable model. The final logistic regression model for burnout used a stepwise selection technique, which was determined to be the most comprehensive method because it combines both forward and backward selection. To justify lumping together different types of clinicians and specialties, 1-way analysis of variance was used to examine if statistically significant differences existed in the means of outcome measures across clinician type (ie, MD, DO, nurse practitioner, or physician assistant) or specialty (ie, primary care, nonprocedural specialist, or procedural specialist). Diagnostics done on the regression and logistic models were the Breusch-Pagan test for constant variance and the Hosmer-Lemeshow test for goodness of fit, noting that P > .05 indicates having constant variance for the regression model and correct fit for the logistic model respectively. (These showed that the models were well calibrated.) Finally, the researchers performed a statistical factor analysis using the varimax rotation method on 9 EHR design and use items to summarize the association of EHRs with stress and burnout. We used SAS version 9.4 (SAS Institute, Inc) for all analyses. Statistical significance was set at P < .05, and all tests were 2-tailed. | III | B |
| Sieja et al [33] | 233 | localized study | not reported | United States | descriptive statistics | II | A |
| Quinn et al [32] | 24 | Physicians with less than 4 years of experience | not reported | United States | Among eligible physicians, 1,763 completed the questionnaire with an unweighted response rate of 61%. | III | B |
| Robinson and Kersey [41] | 3500 | Localized study.  Just-in-time feedback and end-of-day surveys were used to make minor daily adjustments to the training to meet individual participant needs. The feedback included suggestions on the speed of the presentation delivery and clarification of content. | not reported | United States | Relative risks and 95% confidence intervals (CIs) were estimated to test for differences in order set use in 2017 compared with 2015. Descriptive statistics. Statistical analysis was performed using Stata, version 14.2 | III | A |
| Pozdnyakova et al [40] | 6 | The small physician sample size and the short duration of the pilot limited the researchers' abilities to detect a change in burnout and longer term effects on physician and patient satisfaction. The pilot included one 20 to 30-year-old female scribe, and the researchers could not assess the impact of scribe characteristics, such as gender, race, or age. The patient demographics (eg, large percentage of African American patients) may also limit generalizability. To minimize survey burden, the researchers used the single-item burnout question, which may have limited their ability to detect burnout. Moreover, physician time spent on documentation was assessed using self-report, which is subject to recall bias. Lastly, the patient survey was only available in English, and the researchers missed perspectives of non-English speaking patients. | not reported | United States | Research Electronic Data Capture (REDCap) system analyzed surveys. Stata 14 and SAS 9.4 analyzed data. Descriptive statistics and paired t tests were used to analyze data. Logistic regression and generalized linear mixed (GLM) models analyzed patient satisfaction and attitude toward scribes. The GLM models accounted for clustering effects of patient responses within physician. Models initially included the potential covariates of age, gender, and race, and then through the backward model selection procedure, only covariates significantly associated with outcomes were included in final models.  For patient satisfaction, the researchers compared “strongly agree” vs. “agree,” “neutral,” “disagree,” and “strongly disagree,” due to a high baseline level of patient satisfaction. For other outcomes, strongly agree and agree responses were collapsed and analyzed as agree; and strongly disagree, disagree, and neutral were collapsed as disagree. For negative-valence questions, responses were reverse coded so that strongly agree, agree, and neutral were compared to disagree and strongly disagree. Because logistic regression and GLM models produced very similar results, the researchers only presented results from the logistic regression models. | II | B |
| Marmor et al [39] | 39 | none | not reported | United States | descriptive statistics and regression analysis | I | B |
| Denton et al [35] | 20 | semistructured interviews | not reported | United States | descriptive statistics | III | B |
| Kroth et al [38] | 41 | localized study No residents or other trainees were invited | not reported | United States | Text analysis, descriptive statistics | III | B |
| Hauer et al [36] | 1165 | The 2017 study differs from the 2014 and 2009 studies. In addition, the studies were not longitudinal, and it is not known if the 2014 and 2009 respondents participated in the 2017 survey. Localized study. | not reported | United States | Descriptive statistics | III | B |
| Young et al [42] | 982 | Potential weaknesses of the study include the fact that observers had 1 day of training, visits were not recorded to check for intraobserver agreement, time allocation was only kept to the minute (other studies broke down time into 15 second increments), observations included many residents and did not include physicians in private practice, and the ever-present Hawthorne effect. Further analysis (not fully reported here) found that most time or other related differences (eg, number of issues addressed) were explained by differences in patient demographics across the sites. Another weakness is that the times reported for working in the EHR outside of normal clinic hours were estimated, not observed directly. | not reported | United States | Results were analyzed using descriptive statistics. Group comparisons of continuous variables were assessed using independent samples t-tests or analysis of variance (ANOVA) as appropriate, and comparisons of all categorical data were analyzed using chi square. Bivariate correlation coefficients were calculated to identify variables that potentially predicted the time outcomes. Those that had a P value of < .20 were included in the multivariable linear regression models to identify independent predictors of time outcomes. SPSS (Statistical Package for the Social Scientist) version 20 was used for analysis (SPSS Inc., Chicago, IL). All tests were 2-tailed, and alpha levels were set at 0.05 to determine statistical significance. | III | B |
| Khairat et al [37] | 14 | Emergency physicians already have the highest level of burnout | not reported | United States | descriptive statistics | III | B |
| Arndt et al [47] | 142 | Measuring time spent "in" the EHR is not the best measure. Many EHR tasks are associated with multiple subroutines. For example, telephone encounters are often associated with a review of prior notes (Chart Review – Notes) and current medications (Chart Review – Medications) to answer a patient’s question, followed by typing a progress note (Documentation) of the telephone dialog with a patient. As a result, the total time attributed to the Telephone Call category is relatively small given that it captures only the telephone module time and does not account for associated tasks. | not reported | United States | Descriptive statistics | III | B |
| Shahmoradi et al [42] | 90 | questionnaires were used | not reported | Tehran | descriptive statistics | III | B |
| Gregory et al [43] | 6 | Subjective, but not objective, alert workload was related to two of the three dimensions of burnout, including physical fatigue (p = 0.02) and cognitive weariness (p = 0.04), when controlling for organizational tenure. | not reported | United States | descriptive statistics | III | B |
| Jamoom et al [45] | 1471 | While about three-quarters of physicians self-reported their agreement that their practice “optimized its EHR use”, the definition of optimization was left to the respondent's discretion and how physicians interpreted this may be hard to elucidate. | not reported | United States | Descriptive statistics | III | B |
| Reuben et al [46] | 30 | None identified | not reported | United States | The researchers collected time-series data to measure physician efficiency by calculating physicians’ time spent in the examining room during encounters with and without Physician Partners. In addition, physicians were asked to log the amount of time spent on documentation and administrative duties prior to and after a 4-h clinic session and to complete a survey evaluating satisfaction in working with a Physician Partner. Patients were also surveyed about the program. | I | B |
